# Increased Expression of *PS1* Is Sufficient to Elevate the Level and Activity of γ-Secretase *In Vivo*

**DOI:** 10.1371/journal.pone.0028179

**Published:** 2011-11-29

**Authors:** Tong Li, Yue-Ming Li, Kwangwook Ahn, Donald L. Price, Sangram S. Sisodia, Philip C. Wong

**Affiliations:** 1 Department of Pathology, The Johns Hopkins University, School of Medicine, Baltimore, Maryland, United States of America; 2 Department of Neuroscience, The Johns Hopkins University, School of Medicine, Baltimore, Maryland, United States of America; 3 Department of Neurology, The Johns Hopkins University, School of Medicine, Baltimore, Maryland, United States of America; 4 Molecular Pharmacology and Chemistry Program, Memorial Sloan-Kettering Cancer Center, New York, New York, United States of America; 5 Department of Neurobiology, University of Chicago, Chicago, Illinois, United States of America; Thomas Jefferson University, United States of America

## Abstract

Increase in the generation and deposition of amyloid-β (Aβ) plays a central role in the development of Alzheimer's Disease (AD). Elevation of the activity of γ-secretase, a key enzyme required for the generation for Aβ, can thus be a potential risk factor in AD. However, it is not known whether γ-secretase can be upregulated *in vivo*. While *in vitro* studies showed that expression of all four components of γ-secretase (Nicastrin, Presenilin, Pen-2 and Aph-1) are required for upregulation of γ-secretase, it remains to be established as to whether this is true *in vivo*. To investigate whether overexpressing a single component of the γ-secretase complex is sufficient to elevate its level and activity in the brain, we analyzed transgenic mice expressing either wild type or familial AD (fAD) associated mutant *PS1.* In contrast to cell culture studies, overexpression of either wild type or mutant *PS1* is sufficient to increase levels of Nicastrin and Pen-2, and elevate the level of active γ-secretase complex, enzymatic activity of γ-secretase and the deposition of Aβ in brains of mice. Importantly, γ-secretase comprised of mutant PS1 is less active than that of wild type PS1-containing γ-secretase; however, γ-secretase comprised of mutant PS1 cleaves at the Aβ42 site of APP-CTFs more efficiently than at the Aβ40 site, resulting in greater accumulation of Aβ deposits in the brain. Our data suggest that whereas fAD-linked PS1 mutants cause early onset disease, upregulation of PS1/γ-secretase activity may be a risk factor for late onset sporadic AD.

## Introduction

Alzheimer's disease (AD), the most common form of dementia occurring in the elderly, is a progressive neurodegenerative disease characterized pathologically by neuritic (or senile) amyloid plaques and neurofibrillary tangles in the brain [1]. Neuritic plaques are mainly consists of amyloid-β peptides (Aβ) that are generated proteolytically from amyloid-β precursor protein (APP) by the sequential cleavage of β- and γ-secretase. Current genetic and epidemiological studies support the “amyloid cascade hypothesis” of AD [2], which posits that genetic or environmental factors cause abnormal accumulation of dimers, oligomers or higher order assemblies of Aβ, which are neurotoxic and initiate a cascade of events eventually leading to synaptic and neuronal dysfunction and death in cases of AD. This view is supported by the genetic studies in which missense mutations in *APP* [3]–[5], *PS1* and *PS2* [6]–[9], and duplications of *APP* [10] have been linked to cases of early onset familial AD (fAD). All of these mutations either increase the generation of Aβ or increase relative levels of Aβ42, the more toxic form of Aβ. As only symptomatic therapies are currently available, mechanism-based disease modifying therapy remains a major unmet need for AD[11].

FAD accounts for ∼5% of total AD cases, whereas >90% of individuals with AD manifest as late onset sporadic form of AD (sAD). While the genetic risk factors that contribute to Aβ amyloidosis in sAD cases are less clear, putative sporadic cases are influenced by a variety of susceptibility genes and possibly other less well-defined factors [12]. One gene that has been consistently replicated as a major dose-dependent risk factor in a large number of studies across many ethnic groups is the *Apoε4* allele of the *apolipoprotein E* gene (chromosome 19q13), which has been hypothesized to influence Aβ metabolism, Aβ aggregation/degradation/clearance [13]–[15]. Recent research has identified gene variants encoding ubiquilin1 (*UBQLN1*) [16] and sortilin1 (*SORL1*) [17] as risk factors, and GWAS approaches have identified a number of variants (CLU, PICALM and CRI) associated with AD [18], [19], which may also contribute to abnormal APP processing and Aβ accumulation. Therefore, it has been widely accepted that both sporadic and familial AD share the same underlying disease mechanism by promoting the accumulation of Aβ in the brain.

As enzymes required for the generation of Aβ, increase of β- and γ-secretase activities in the brain represent potential risks for the development of AD. While it has been reported that higher levels of BACE1 are associated with increased risk for AD [20], [21], less information is available regarding the relationships between altered levels of γ-secretase and risk of development of AD. Moreover, the idea that γ-secretase is a risk factor for AD is challenged by the assumption that upregulation of γ-secretase is difficult to achieve. Although PS alone exhibits γ-secretase activity, each component of the γ-secretase complex, PS, Nct, APH-1 and PEN-2, is absolutely required for γ-secretase activity *in vivo* [22]. While the mechanism of assembly of γ-secretase complex is still not completely understood, the accumulation of γ-secretase appears to be tightly controlled. A simple model of γ-secretase assembly invokes initially the formation of a relatively stable pre-complex comprised of Nct and Aph-1 [23], and the subsequent associations of PS and PEN-2 with this pre-complex lead to the formation of the mature γ-secretase complex. The mature γ-secretase complex is characterized by the post-translational modifications of various subunits, while the enzyme complex is transported en route to the plasma membrane. Evidence indicates that some components of γ-secretase complex, such as PS, are not stable unless they are associated with other components to form the pre- or mature γ-secretase complex [23]–[26]. Therefore, the downregulation of γ-secretase can be achieved by reducing anyone of the four essential components of γ-secretase.

In contrast, overexpression of any one member has little effect on the levels of other components or the overall γ-secretase activity in cell culture systems [27]–[31]. In fact, overexpression of even any combination of three proteins is still not sufficient to increase the activity of γ-secretase; elevation in γ-secretase activity can only be achieved by overexpressing all four proteins [32]. These observations led to the assumption that the upregulation of γ-secretase is unlikely to occur in the brain since upregulation of all four components of γ-secretase is necessary to increase the level of γ-secretase. However, this assumption is based on *in vitro* cell culture studies and has not been thoroughly tested *in vivo*.

In this report, we document that, in contrast to the observations derived from cell culture studies, increased expression of *PS1* alone significantly increased the level and activity of γ-secretase and the Aβ burden in the brain, suggesting the possibility that elevation of γ-secretase may be a risk factor in AD.

## Results

**Figure 1:**
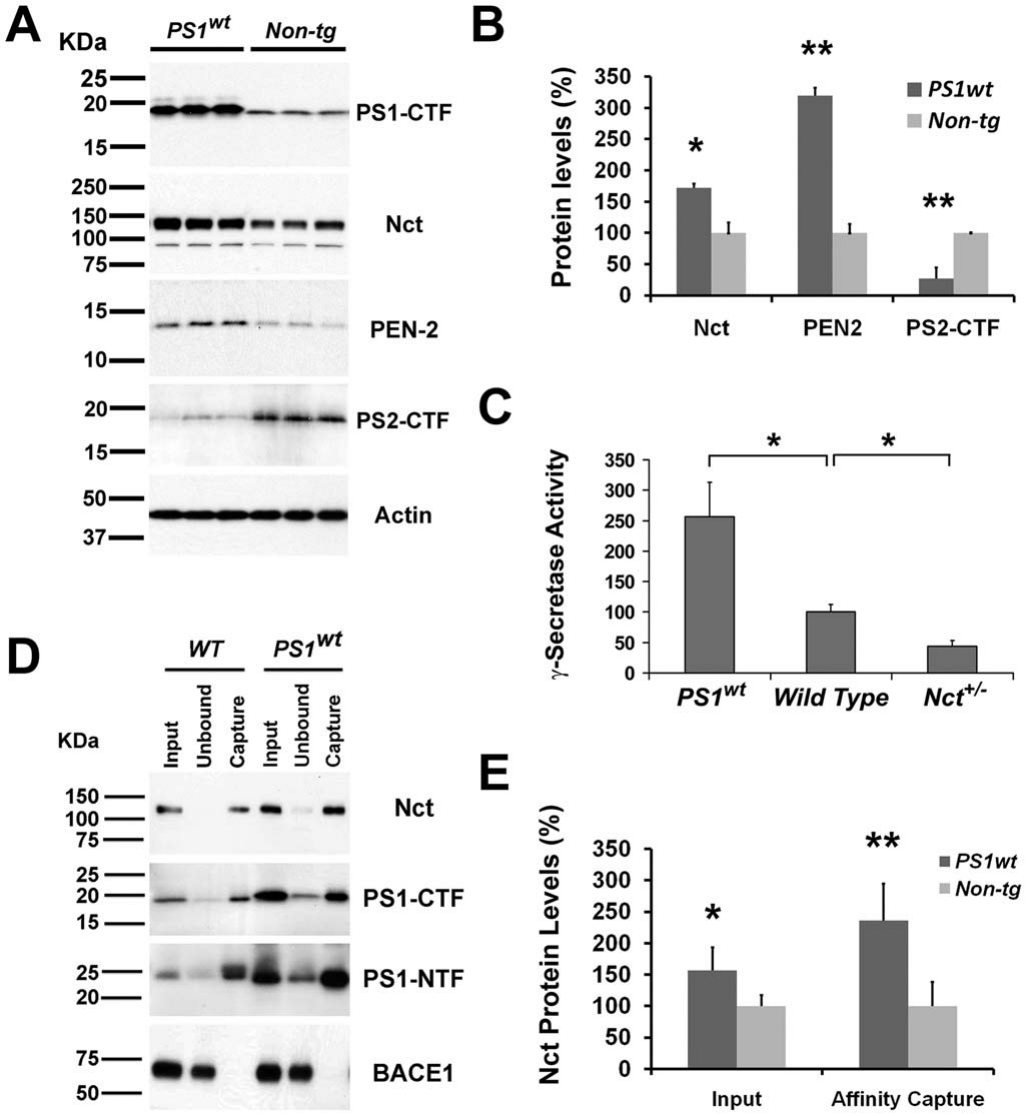
Increase of γ-secretase activity in brains of *PS1* mice. (A) Protein extracts (20 µg each) from brains of *PS1* transgenic (lanes 1–3) and non-transgenic mice (lanes 4–6) were immunoblotted with anti-sera specific to Nct, PS1-CTF, PEN2, PS2-CTF, or Actin. (B) Quantification of signals of Nct, Pen-2, and PS2-CTF in protein blots by Image J program. The signal density was normalized using Actin signals derived from the same blot. (C) In vitro γ-secretase assay of brain extracts of wild type, *PS1* transgenic, and *Nct^+/−^* mice. The data were average from 4 samples. (D) The CHAPSO solubilized membrane proteins from wild type and *PS1* transgenic mice were incubated with Compound 4 followed by precipitation with streptavidin-coupled beads. Solubilized membranes before ligand addition (Input: 10% of total), captured fraction (Capture*:* 20% of total) of the affinity ligand and the corresponding supernatants after capture (Unbound: 7.5% of total) were analyzed by Western blot using antisera specific to Nct, PS1-CTF, PS1-NTF, or BACE1. (E) Quantification of signals of Nct levels from in protein blots by Image J program. The signal density was normalized using BACE1 signals derived from the same blot.

While previous efforts demonstrated that increased expression of all four components of γ-secretase is required to upregulate its enzymatic activity in cultured cells, it remains to be established whether overexpression of all four components is necessary to increase the level of γ-secretase *in vivo*. To determine whether overexpression of a single component of the γ-secretase is sufficient to elevate its level and activity in the brain, we analyzed transgenic mice expressing human wild type *PS1* under the control of mouse *PrP* promoter [33]. Initial protein blot analysis of levels of PS1 in brains of *PS1* transgenic mice using antibodies specific to C-terminal fragments of PS1 (one of two processed fragments of PS1 associated with the mature γ-secretase complex), revealed overexpression of human PS1 protein (Fig. 1A). To confirm the “replacement effect” of the exogenous human PS1, we assessed the level of endogenous PS by analyzing the level of PS2 using antiserum recognizing the C-terminal fragment of PS2 (PS2-CTF). As expected, the level of PS2-CTF was significantly reduced in brains of *PS1* transgenic mice as compared to that of controls (Fig. 1A and 1B). Since anti-PS1 antibody can recognize the human and mouse PS1 with higher affinity, levels of γ-secretase complex in *PS1* transgenic mice cannot be evaluated by western blot using antibodies against PS1. To assess the levels of γ-secretase complex, we analyzed the endogenous protein levels of Nct and PEN2, two key components of γ-secretase complex in brains of *PS1* transgenic mice. Interestingly, protein levels of PEN2 was significantly increased (∼200%, p<0.005) in *PS1* transgenic mice as compared to that of wild type mice (Fig. 1A and 1B). Consistent with this finding, we also observed an increase of endogenous Nct in *PS1* transgenic mice as compared to that of wild type mice (Fig. 1A and 1B).

To determine whether the γ-secretase complex in *PS1* transgenic mice is functional and active, we used an *in vitro* γ-secretase activity assay to assess the γ-secretase enzymatic activity in protein extracts derived from brains of *PS1* transgenic mice. Interestingly, γ-secretase activity at both the Aβ40 (Fig 1C) and Aβ42 cleavage site in brain extracts of *PS1* transgenic mice increased ∼150% as compared to control mice, corroborating the increase in protein levels of components of the γ-secretase complex observed in the brains. Consistent with our previous studies [34], [35], γ-secretase activities in brains of *Nct^+/−^* mice were reduced ∼50% as compared with that of wild type mice (Fig. 1C). These results establish that elevated levels of *PS1* alone in the brain of mice is sufficient to increase the level and enzymatic activity of mature γ-secretase complex *in vivo*.

While both PS1 and PS2 can form functional γ-secretase complex, it has been reported that the specific activity of PS1-containing γ-secretase is higher than that of PS2- containing complex *in vitro* [36]. Exogenous expression of human PS1 replaces both the endogenous PS1 and PS2, thus most γ-secretase in the *PS1^wt^* transgenic mice are human PS1 containing γ-secretase complex. While we observed a significant increase of γ-secretase activity in *PS1^wt^* mice, it is possible that the increased γ-secretase activity in *PS1^wt^* transgenic mice is due to relative abundance of PS1-containing γ-secretase complex. To examine whether overexpression of *PS1* can increase the levels of active γ-secretase complex, we used a well characterized γ-secretase transition-state analogue inhibitor based probe (compound 4) [37] to quantify the active fraction of γ-secretase *in vivo*. Biotin labeled compound 4 can specifically bind to the active γ-secretase complex, thus active γ-secretase can be captured using streptavidin-labeled bead [37]. Membrane proteins were extracted using buffer containing CHAPSO and the levels of PS1 and Nct in unbounded fractions and affinity captured fractions of membrane protein extracts were subjected to immunoblot analysis using antisera against Nct, PS1-NTF and PS1-CTF. We found that ∼70% of CHAPSO extracted γ-secretase complex can be captured by the compound-4, while another membrane-bound secretase, BACE1, was not captured by compound 4 (Fig. 1D), which confirmed that compound 4 is a specific and high affinity inhibitor of γ-secretase. Comparing the levels of Nct and PS1 in the affinity captured fraction of *PS1^wt^* transgenic with that of non-transgenic control mice, we found that the levels of Nct in affinity-captured fraction of *PS1^wt^* mice increased by more than 130% (Fig. 1D and 1E). These data further support our view that overexpression of *PS1* alone in the brain increase the protein level of mature γ-secretase complex and enzymatic activity of γ-secretase *in vivo*.

**Figure 2:**
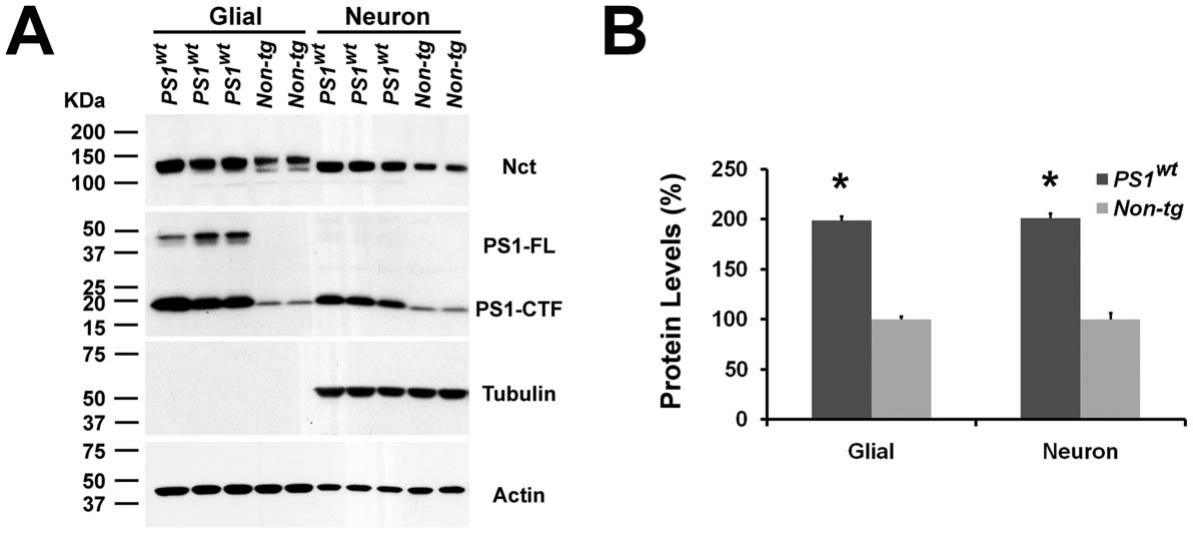
Increase of γ-secretase in primary neurons and non-neuronal cells derived from *PS1* mice. (A) Protein extracts (20 µg each) from primary neuronal and non-neuronal cultures derived from brains of *PS1* and non-transgenic control mice were immunoblotted with anti-sera specific to Nct, PS1-CTF, β-Tubulin III or Actin. (B) Quantification of Nct signals in protein blot of primary neuronal and non-neuronal cell extracts by Image J program. The signal density was normalized using Actin signals derived from the same blot.

While our studies demonstrate that overexpression of PS1 can significantly increase the level of mature γ-secretase in the brain, it is not clear whether such an increase is derived from neuronal or non-neuronal cells. Since Aβ is generated from sequential cleavage of APP by β- and γ-secretase and β-secretase (BACE1) is abundantly expressed in neurons [38], the magnitude of Aβ secretion will be dependent on the level of γ-secretase in neuronal cells. To determine whether overexpression of *PS1* can upregulate the level of γ-secretase in neurons, we isolated primary neuronal and non-neuronal cells from brains of *PS1* transgenic and wild type embryos (at embryonic day 17) and assessed the levels of γ-secretase in these cells. The purity of our neuronal cultures was determined using the neuron specific marker, β-tubulin III (Fig. 2A). Interestingly, in either the neuronal or non-neuronal cell cultures derived from *PS1* transgenic mice, the levels of Nct were significantly increased (Fig. 2A and 2B), indicating that the overexpression of *PS1* alone is sufficient to increase the level of γ-secretase in either neuron or non-neuronal cells.

**Figure 3:**
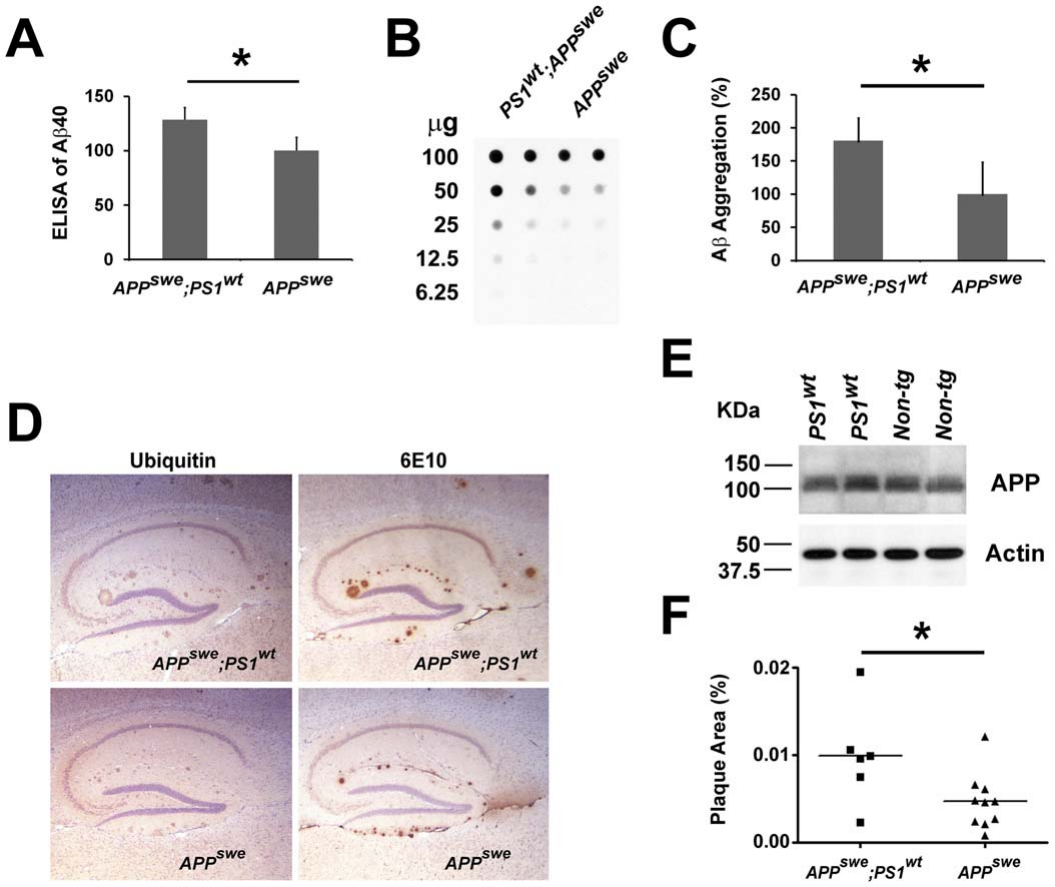
Increase in generation and deposition Aβ in the brains of *APP^swe^;PS1* mice. (A) ELISA analysis of Aβ40 peptides in the protein extracts of brains of *PS1* and non-transgenic control mice. The data were average +/− SEM from 5 mice for each genotype. (B) Sagittal brain sections (10 µm) of hippocampus area of 22-months old *APP^swe^;PS1* and *APP^swe^* female mice. The Aβ plaques were visualized by immunostaining with antibodies specific to ubiquitin and Aβ peptides (6E10). (C) Quantitative analysis of the levels of Aβ aggregation in the brains of *APP^swe^;PS1* and *APP^swe^* mice at 22 months of age by filter trap assay. (D) Quantification of the signals of Aβ aggregations in the filter trap assay using Image J program. (E) Analysis of Aβ deposition using unbiased stereology in the hippocampus of 22-months old *APP^swe^;PS1* (n = 6) and *APP^swe^* (n = 10) female mice.

To examine the effects of upregulation of γ-secretase in *PS1* transgenic mice on generation of Aβ and Aβ amyloidosis, we crossed the *PS1* mice with a mouse model of amyloidosis, *APP^swe^* transgenic mice [39], to generate *APP^swe^;PS1^wt^* mice. Although no Aβ deposits were detected in the brains of 9 month-old *APP^swe^;PS1^wt^* or *APP^swe^* mice, ELISA analysis showed an ∼30% increase in the level of Aβ40 in brains of *APP^swe^;PS1^wt^* mice as compared to that of *APP^swe^* mice (Fig. 3A, p<0.05), indicating that upregulation of *PS1* alone is sufficient to increase the generation of Aβ in brains of mice. To determine whether such increase in γ-secretase activity would accelerate amyloid deposition, serial brain sections of 22 month-old *APP^swe^;PS1^wt^* mice were stained with antibodies against Aβ (6E10) or against ubiquitin. Interestingly, greater amount of Aβ plaques were observed in brain sections of *APP^swe^;PS1^wt^* mice as compared to that of *APP^swe^* mice (Figs. 3D). Since the levels of APP in *APP^swe^;PS1^wt^* mice is identical to that of *APP^swe^* mice (Fig. 3E), the elevation of Aβ deposition is likely due to an increase in levels of γ-secretase in brains of *PS1^wt^* mice. To quantify the levels of Aβ deposition, we used both filter trap and unbiased stereology approaches to assess the amyloid burden in the brains of these transgenic mice. We observed not only an ∼2 fold increase of aggregated Aβ (Fig. 3B and 3C), but importantly, a corresponding elevation of amount of Aβ plaques (Fig. 3F) in the brains of *APP^swe^;PS1^wt^* mice as compared to those of *APP^swe^* mice. These results demonstrate that overexpressing *PS1* alone is sufficient to increase the activity of γ-secretase and deposition of Aβ in the brain and thus suggest that such increase of γ-secretase activity may confer risk in development of AD.

**Figure 4:**
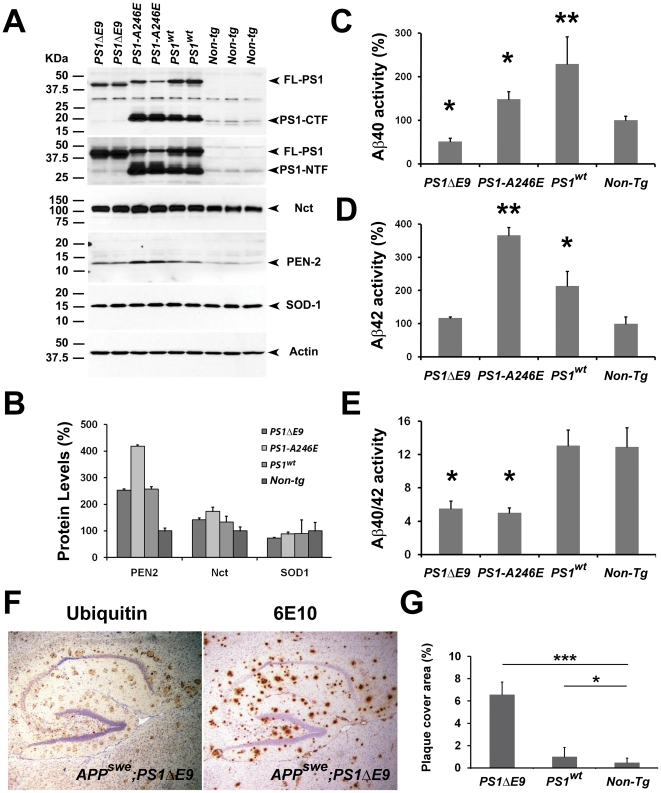
Overexpression of FAD linked *PS1* mutant*s* increases the level of γ-secretase in the brain. (A) Protein extracts (20 µg each) from brains of *PS1ΔE9* (lanes 1, 2), *PS1-A246E* (lanes 3, 4), *PS1wt* (lanes 5, 6) and non-transgenic mice (lanes 7–9) were immunoblotted with anti-sera specific to Nct, PS1-NTF, PS1-CTF, PEN2, SOD-1 or Actin. (B) Quantification of signals of Nct, PEN2 and SOD-1 in protein blots of mutant and wild type *PS1* and non-transgenic control mice by Image J program. The signal density was normalized using Actin signal derived from the same blot. (C) *In vitro* γ-secretase assay of Aβ40 cleavage in brain extracts of *PS1ΔE9*, *PS1-A246E*, *PS1^wt^* and non-transgenic control mice. The data represent averages +/- SEM from 4 mice for each genotype. (D) *In vitro* γ-secretase assay of Aβ42 cleavage in brain extracts of *PS1ΔE9*, *PS1-A246E*, *PS1^wt^* and non-transgenic control mice. The data represent averages +/− SEM from 4 mice for each genotype. (E) The ratio of Aβ40 and Aβ42 cleavage activity in brain extracts of *PS1ΔE9*, *PS1-A246E*, *PS1^wt^* and non-transgenic control mice. (F) Sagittal brain sections (10 µm) of 22-months old *APP^swe^;PS1ΔE9* female mice. The Aβ plaques were visualized by immunostaining with antibodies specific to ubiquitin and Aβ peptides (6E10). (G) Analysis of Aβ deposition using unbiased stereology in brain sections of 22-months old *APP^swe^;PS1ΔE9* (n = 5), *APP^swe^;PS1* (n = 6), and *APP^swe^* (n = 10) female mice. The data represent averages +/− SEM from each genotype.

To further establish that overexpression of *PS1* alone could increase the level of γ-secretase in the brain, we examined the effects of overexpression of a variety of fAD-linked *PS1* mutants in mice. We first examined the protein levels of γ-secretase in transgenic mice that overexpress *PS1ΔE9* and *PS1-A246E* (Fig. 4A). Consistent with our observation in wild type *PS1* transgenic mice, mice expressing *PS1* mutants also exhibit significant increase in the level of γ-secretase complex in the brains. The levels of several components of γ-secretase (PS1, Nct, PEN2) in *PS1ΔE9* were similar as compared with those of *PS1* transgenic mice, while the levels of components in *PS1-A246E* mice were higher than those of *PS1* transgenic mice (Fig. 4B), which is consistent with previous reports [40]. Surprisingly, the increase in levels of γ-secretase components in the brains of *PS1ΔE9* and *PS1-A246E* mice did not lead to significant increase in cleavage activity at the Aβ40 site (Fig. 4C). Rather, the γ-secretase activity at the Aβ40 site in brains of *PS1-A246E* mice were 2–3 folds lower than that of wild type *PS1* mice (Fig. 4C) and Aβ40 site cleavage activity observed in *PS1ΔE9* mice was even lower than that of non-transgenic mice (Fig. 4C). In parallel, we assessed the cleavage activity at the Aβ42 site in brain extracts derived from these mice (Figure 4D). Interestingly, the activity at the Aβ42 site of γ-secretase in *PS1-A246E* mice was significantly higher than that of *PS1* and non-transgenic mice (Fig. 4D). γ-Secretase activity in brains of *PS1ΔE9* mice was similar to that of non-transgenic mice. These findings indicate that the γ-secretase complex containing mutant *PS1* is less active than that comprised of wild type *PS1*, especially with respect to the cleavage activity at the Aβ40 site. However, whereas the ratio of Aβ40/Aβ42 activity in *PS1* mice was identical to that of non-transgenic mice, this ratio was significantly decreased in both *PS1ΔE9* and *PS1-A246E* mice (Fig. 4E). These observations are consistent with the view that γ-secretase containing fAD-linked *PS1* mutants elevate the relative cleavage activity at the Aβ42 site as compared to the Aβ40 site [39].

To compare the effects of mutant *PS1* on Aβ deposition with that of wild type *PS1*, we crossed *PS1ΔE9* mice with the *APP^swe^* mice to generate *PS1ΔE9*;*APP^swe^* mice and assessed the Aβ amyloidosis in their brains. As anticipated, we observed that the Aβ burden in the brains of *PS1ΔE9;APP^swe^* mice was significantly higher than that of the *APP^swe^* mice or *PS1^wt^;APP^swe^* mice (Fig. 4F and 4G). These results are consistent with the idea that the relative ratio of Aβ42/Aβ40 is a critical determinant for Aβ deposition in the brain [39].

## Discussion

Since γ-secretase is a key enzyme for the generation of Aβ peptides, understanding how γ-secretase contributes to the development of AD has been a major focus in the field. Mutations in *PS1* and *PS2* have been identified in some rare cases of early onset FAD [6]–[9]. These mutations increase the relative levels of Aβ42 [39], a more amyloidogenic and toxic form of Aβ. In contrast, that increases in level of γ-secretase accelerate the production of Aβ to elicit the Aβ associated pathology would provide a molecular mechanism for increasing the risk for AD. Duplications of *APP* [10] in cases of AD is an example of such a mechanism in which risk for AD is elevated through the increased production of Aβ. However, no genetic association between increased level of γ-secretase and higher risk of AD has been reported. Furthermore, it is unknown as to how expression and accumulation of γ-secretase is regulated in the brain. It is well recognized that the subunit assembly of γ-secretase complex is tightly regulated such that excess subunits like PS or PEN2, which are not recruited into stable complexes are rapidly degraded. Therefore, deletion of any component of the γ-secretase leads to the disassembly of the γ-secretase complex. In contrast, cell culture studies showing that levels of γ-secretase can only be elevated by increased expression of all four components has fueled the idea that γ-secretase would be more amenable to mechanisms that down-regulates its levels than those that up-regulates it. For example, a mutation that reduces expression of one of the four components is predicted to decrease the level of γ-secretase. However, even polymorphism occurring in three of the four genes would still not be sufficient to increase γ-secretase activity, only variants occurring simultaneously in all four genes encoding components of the γ-secretase may up-regulate its activity. The realization that γ-secretase is an essential enzymes for not only the processing of APP, but also required for processing a growing list of type I transmembrane proteins in a process called “regulated intramembrane proteolysis” [41] indicates that maintenance of a stable level of γ-secretase is essential for the normal function of organisms. Surprisingly, the studies described herein demonstrate that increased expression of *PS1* alone is sufficient to increase the γ-secretase activity and to elevate deposition of Aβ *in vivo*, indicating that alteration of a single component of γ-secretase is sufficient to elevate the level of γ-secretase. While the clinical relevance of our finding is still unclear, our observation also suggests that increase in levels of γ-secretase could be a potential risk factor in AD. Although no association between risk of AD and elevated levels of γ-secretase has been documented in cases of AD, increased levels in PS1 has been reported in senescence-accelerated mice (SAMP8); it is possible that increased levels of PS1 may lead to aberrant accumulation of Aβ and loss of memory in these mice [42].

Although we showed increased expression of PS1 is sufficient to elevate γ-secretase *in vivo*, it appears that increased expression of *Nct* and *Aph-1* does not elevate the level of γ-secretase [43]. While the effects of overexpressing *PEN2* on the levels of γ-secretase has not been tested *in vivo*, the fact that the active center of γ-secretase complex resides within the PS subunit [22] would support the idea that PS is the limiting factor that governs the assembly of γ-secretase *in vivo*. Interestingly, in cells lacking either Nct, Aph-1 or Pen-2 [24], [25], [44], PS1 consistently appears to be the least stable subunit as compared to other components. In contrast, without PS, the amount of Aph1-Nct pre-complex is not significantly affected in cells, although only immature form of Nct remains. That PEN-2 is less stable than Nct or Aph-1 but more stable than PS as remnants of Pen2 still can be detected in *PS* or *Nct* null cells would support the notion that alterations in levels of PS1 should have the most dramatic impact on the levels of γ-secretase *in vivo*.

While the increase of γ-secretase elevated the production of Aβ in mouse brains of *PS1* mice, the amyloid burden was only modestly increased. In parallel, the level of γ-secretase in the fAD-linked mutants *PS1-A246* and *PS1-ΔE9* mice can also be increased. Interestingly, the γ-secretase complex containing *PS1-A246E* or *PS1-ΔE9* mutant exhibited a drastic reduction in level of γ-secretase activity at the Aβ40 site as compare to that of wild type *PS1* mice. Instead, relative levels for Aβ42 processing were increased as compared to that of the wild type *PS1* mice whose Aβ42/40 ratio remained constant and consequently, accelerated the amyloid burden in brains of *PS1-ΔE9* mice. Our findings are consistent with the notion that Aβ42 is more toxic and readily form Aβ aggregates in the brain [39].

In summary, our data support the view that overexpression of *PS1* in vivo is sufficient to increase the generation and deposition of Aβ in the brain. Importantly, this unexpected finding strongly implicates that upregulation of γ-secretase could contribute to increased risk of AD, especially in late onset sporadic AD.

## Materials and Methods

### Animals and cell lines

*PS1(wt)*, *PS1ΔE9*, *PS1A246E*, and *APP^swe^* mice were generated as described previously [33], [45]. *PS1(wt)* and *PS1ΔE9*, mice were crossed with *APP^swe^* mice to generate *PS1wt*;*APP^swe^* and *PS1ΔE9;APP^swe^* mice. Animal housing and experimental protocols were approved by the Animal Care and Use Committee of the Johns Hopkins University (Protocol # MO09M243).

### Affinity Capture of Endogenous γ-Secretase

The affinity capture of active γ-secretase was performed using a procedure modified from that described previously [37]. In short, 100 µg CHAPSO-solubilized membrane proteins were incubated for 2 h at 37°C in 50 mM PIPES, pH 7.0, 150 mM KCl, 5 mM CaCl_2_, 5 mM MgCl_2_, and protease inhibitors in 0.5% (v/v) CHAPSO with 20 nM biotinylated affinity probe (compound 4), which is modified from a potent transition state analog of γ-secretase (L458) [37]. 250 µl (10 mg/mL) of streptavidin-coupled magnetic beads (Dynal, Invitrogen) was added to the reaction and incubated overnight at 4°C. Captured complexes were washed five times with TBST buffer (Tris-buffered saline containing 0.1% (v/v) Tween 20) and eluted with 2X SDS sample buffer. Samples were resolved by SDS-PAGE and transferred to a polyvinylidene difluoride membrane. PS1 and Nct were detected by protein blotting using antiserum against PS1-NTF, PS1-CTF, and Nct. The levels of BACE1 served as a loading control. In all cases, protein blots shown are representatives of three or more experiments.

### Primary Cortical Neuronal and Non-neuronal Cells

Mouse primary neuronal cultures were obtained from cerebral cortices of E18 embryos in a procedure modified from that described previously [46]. In Short, brains were collected from E18 PS1 transgenic or non-transgenic embryos and kept in ice-cold Hanks' balanced salt solution (HBSS). The dissected cortices were minced, digested with an enzyme solution containing 10U of papain (Sigma) and 10 µg/ml DNase I (Sigma) for 20 minutes, and cells were mechanically dissociated in culture medium with pipettes. Cells were counted and plated onto poly-l-lysine and laminin coated six-well plates in Neurobasal medium supplemented with 2% B27, 0.5 mM l-glutamine, 100 U/ml penicillin and 100 µg/ml streptomycin (all from Invitrogen). Medium was replaced with fresh warm medium two hours later. Four days after plating, 2.5 µM cytosine arabinoside (Ara-C; Sigma) was added to eliminate proliferating astrocytes in the cultures. After 2 days of Ara-C exposure, the medium was changed once before the cultures were subjected to further analysis. To derive non-neuronal cells (astrocytes, microglia, and oligodendrocytes), dissected cortices from the brains were minced on 35-mm petri dishes on ice. To separate the cells, the minced tissues were suspended in 750 µl 0.25% trypsin/EDTA containing 1 mg/ml DNase I and incubated at 37°C for 15 min. Cells were centrifuged for 5 min at 300×*g* at room temperature and the cell pellets were dissociated in 5 ml of MEM containing 20% FBS.

### Immunoblot and Antibodies

Immediately after euthanasia, mouse brain tissues were dissected and proteins were extracted with TEPER buffer (Pierce Chemical Co., Rockford, IL) containing complete protease inhibitor cocktail (Roche, Indianapolis, IN). The protein concentrations in the supernatants were determined by the BCA method (Pierce Chemical Co., Rockford, IL) and equal amount of protein lysates resolved on 4–20% Tris-Glycine SDS PAGE gels, transferred to polyvinylidene difluoride (PVDF) (Invitrogen, Carlsbad, CA) membranes, and probed with following antibodies: anti-Nicastrin [24]; antisera specific for PS1 [47], anti-PEN-2 [48], and anti-Aph-1aL antibody (Covance Inc, Princeton, NJ). Blots were probed using monoclonal antibody against actin and rabbit anti-β-tubulinIII antisera (Sigma) were used as loading control. Immunoblots were developed using enhanced chemiluminescence method (Millipore Corp. MA).

### In vitro γ-secretase activity assay

The γ-secretase activity of mouse brains were performed as described previously [49]. After mouse brain membranes were extracted in buffer A (50 mM Mes, pH 6.0/5 mM MgCl_2_/5 mM CaCl_2_/150 mM KCl) with 1×complete protease inhibitor mixture (Boehringer Mannheim), 8 µg of membrane protein was incubated with APP recombinant substrate (1 µM) in the presence of 0.25% CHAPSO in buffer B (50 mM Pipes, pH 7.0/5 mM MgCl_2_/5 mM CaCl_2_/150 mM KCl) at 37°C. The reactions were stopped by adding RIPA (150 mM NaCl/1.0% NP-40/0.5% sodium deoxycholate/0.1% SDS/50 mM Tris HCl, pH 8.0) and assayed with G2-10 antibody, which recognizes the C-terminus of Aβ40 by ECL.

### Filter Trap Assay

Cellulose acetate membranes with 0.2 µm pore size (OE66, Schleicher & Schuell, Keene, NH) were used to trap the aggregates containing Aβ from the brain lysate [50]. Briefly, mouse brains were weighed and then homogenized in 10 volumes of PBS (pH 7.4) containing a protease inhibitor cocktail (Roche, Indianapolis, IN). Homogenates were centrifuged at 3,000 rpm for 5 min at 4°C in a microcentrifuge. The protein concentrations in the supernatants were determined by the BCA method (Pierce Chemical Co., Rockford, IL). Before filtering, the samples were diluted with PBS (100–200 µL) and adjusted to a final concentration of 1% SDS. After filtering, using a 96-well dot-blot apparatus (Bio-Rad Laboratories, Hercules, CA), the membranes were washed twice with 500 µL of PBS (pH 7.4) per well. Proteins trapped by the filter were detected by rabbit anti-Aβ antiserum (Invitrogen, South San Francisco, CA) following conditions used for immunoblotting analysis.

### Aβ ELISA Assay

To measure the Aβ levels *in vivo*, the brains of *APP^swe^;PS1* and *APP^swe^* mice were dissected on ice, and homogenized in PBS buffer containing 1% triton X-100 and complete protease inhibitor cocktail. After the lysates were centrifuged at 100,000×g for 30 min, the supernatants containing soluble Aβ peptides were collected for assay. The concentrations of total protein extracts were determined by the BCA method (Pierce Chemical Co., Rockford, IL). Aβ40 levels were measured using a quantitative sandwich ELISA kit (Invitrogen, South San Francisco, CA) that specifically detects human Aβ.

### Histology and Immunohistochemical Analysis

For histological and immunohistochemical analysis of Aβ amyloidosis, mice were perfused with 4% PFA in PBS. Brains were removed, embedded in paraffin, sectioned and processed. Sections were analyzed immunohistochemically by the peroxidase-antiperoxidase method using antibodies specific for: Aβ (6E10)(Signet Laboratories, Inc. Dedham, MA) and ubiquitin (Dako Cooperation, Carpinteria, CA).

### Measurement of amyloid plaques in mouse brain by unbiased stereology

To assess the Aβ plaque load, one half of the sagitally bisected mouse brain was immersion fixed in 4% of PFA and embedded in paraffin. The brains were sectioned at 10 µm and sections were selected at 8-section intervals for analysis using a light microscope interfaced with a Stereo Investigator (MicroBrightfield, Inc.). The quantitative analysis was based on area fraction of ubiquitin immunoreactivity as described previously [51].

### Statistical analysis

All data were analyzed statistically by student's t-test or ANOVA. In all tests, the level of significance was at p<0.05.

## References

[1] Wong PC, Li T, Price DL, Brady ST, Siegel GJ, Albers RW (2006). Neurobiology of Alzheimer's Disease.. Basic Neurochemistry.

[2] Hardy J, Selkoe DJ (2002). The amyloid hypothesis of Alzheimer's disease: progress and problems on the road to therapeutics.. Science (New York, N Y.

[3] Goate A, Chartier-Harlin M-C, Mullan M, Brown J, Crawford F (1991). Segregation of a missense mutation in the amyloid precursor protein gene with familial Alzheimer's disease.. Nature.

[4] Murrell J, Farlow M, Ghetti B, Benson MD (1991). A mutation in the amyloid precursor protein associated with hereditary Alzheimer's disease.. Science (New York, N Y.

[5] Naruse S, Igarashi S, Kobayashi H, Aoki K, Inuzuka T (1991). Mis-sense mutation Val->Ile in exon 17 of amyloid precursor protein gene in Japanese familial Alzheimer's disease.. Lancet.

[6] Rogaev EI, Sherrington R, Rogaeva EA, Levesque G, Ikeda M (1995). Familial Alzheimer's disease in kindreds with missense mutations in a gene on chromosome 1 related to the Alzheimer's disease type 3 gene.. Nature.

[7] Sherrington R, Rogaev EI, Liang Y, Rogaeva EA, Levesque G (1995). Cloning of a gene bearing missense mutations in early-onset familial Alzheimer's disease.. Nature.

[8] Levy-Lahad E, Wasco W, Poorkaj P, Romano DM, Oshima J (1995). Candidate gene for the chromosome 1 familial Alzheimer's disease locus.. Science (New York, N Y.

[9] Levy-Lahad E, Wijsman EM, Nemens E, Anderson L, Goddard KAB (1995). A familial Alzheimer's disease locus on chromosome 1.. Science (New York, N Y.

[10] Rovelet-Lecrux A, Hannequin D, Raux G, Meur NL, Laquerriere A (2006). APP locus duplication causes autosomal dominant early-onset Alzheimer disease with cerebral amyloid angiopathy.. Nat Genet.

[11] Golde TE, Schneider LS, Koo EH (2011). Anti-abeta therapeutics in Alzheimer's disease: the need for a paradigm shift.. Neuron.

[12] Bertram L, Lill CM, Tanzi RE (2010). The genetics of Alzheimer disease: back to the future.. Neuron.

[13] DeMattos RB, Cirrito JR, Parsadanian M, May PC, O'Dell MA (2004). ApoE and Clusterin Cooperatively Suppress Abeta Levels and Deposition. Evidence that ApoE Regulates Extracellular Abeta Metabolism In Vivo.. Neuron.

[14] Bu G (2009). Apolipoprotein E and its receptors in Alzheimer's disease: pathways, pathogenesis and therapy.. Nat Rev Neurosci.

[15] Kim J, Basak JM, Holtzman DM (2009). The role of apolipoprotein E in Alzheimer's disease.. Neuron.

[16] Bertram L, Hiltunen M, Parkman M, Ingelsson M, Lange C (2005). Family-Based Association between Alzheimer's Disease and Variants in UBQLN1.. New England Journal of Medicine.

[17] Rogaeva E, Meng Y, Lee JH, Gu Y, Kawarai T (2007). The neuronal sortilin-related receptor SORL1 is genetically associated with Alzheimer disease.. Nat Genet.

[18] Lambert JC, Heath S, Even G, Campion D, Sleegers K (2009). Genome-wide association study identifies variants at CLU and CR1 associated with Alzheimer's disease.. Nat Genet.

[19] Harold D, Abraham R, Hollingworth P, Sims R, Gerrish A (2009). Genome-wide association study identifies variants at CLU and PICALM associated with Alzheimer's disease.. Nat Genet.

[20] Li R, Lindholm K, Yang LB, Yue X, Citron M (2004). Amyloid beta peptide load is correlated with increased beta-secretase activity in sporadic Alzheimer's disease patients.. Proc Natl Acad Sci U S A.

[21] Hebert SS, Horre K, Nicolai L, Papadopoulou AS, Mandemakers W (2008). Loss of microRNA cluster miR-29a/b-1 in sporadic Alzheimer's disease correlates with increased BACE1/beta-secretase expression.. Proc Natl Acad Sci U S A.

[22] Ahn K, Shelton CC, Tian Y, Zhang X, Gilchrist ML (2010). Activation and intrinsic {gamma}-secretase activity of presenilin 1.. Proc Natl Acad Sci U S A.

[23] Shirotani K, Edbauer D, Kostka M, Steiner H, Haass C (2004). Immature nicastrin stabilizes APH-1 independent of PEN-2 and presenilin: identification of nicastrin mutants that selectively interact with APH-1.. J Neurochem.

[24] Li T, Ma G, Cai H, Price DL, Wong PC (2003). Nicastrin Is Required for Assembly of Presenilin/gamma -Secretase Complexes to Mediate Notch Signaling and for Processing and Trafficking of beta -Amyloid Precursor Protein in Mammals.. J Neurosci.

[25] Ma G, Li T, Price DL, Wong PC (2005). APH-1a is the principal mammalian APH-1 isoform present in gamma-secretase complexes during embryonic development.. J Neurosci.

[26] Prokop S, Shirotani K, Edbauer D, Haass C, Steiner H (2004). Requirement of PEN-2 for stabilization of the presenilin N-/C-terminal fragment heterodimer within the gamma-secretase complex.. J Biol Chem.

[27] Edbauer D, Winkler E, Regula JT, Pesold B, Steiner H (2003). Reconstitution of gamma-secretase activity.. Nat Cell Biol.

[28] Takasugi N, Tomita T, Hayashi I, Tsuruoka M, Niimura M (2003). The role of presenilin cofactors in the gamma-secretase complex.. Nature.

[29] Kimberly WT, LaVoie MJ, Ostaszewski BL, Ye W, Wolfe MS (2003). Gamma-secretase is a membrane protein complex comprised of presenilin, nicastrin, Aph-1, and Pen-2.. Proc Natl Acad Sci U S A.

[30] Kim SH, Ikeuchi T, Yu C, Sisodia SS (2003). Regulated hyperaccumulation of presenilin-1 and the "gamma-secretase" complex. Evidence for differential intramembranous processing of transmembrane subatrates.. J Biol Chem.

[31] Farmery MR, Tjernberg LO, Pursglove SE, Bergman A, Winblad B (2003). Partial Purification and Characterization of {gamma}-Secretase from Post-mortem Human Brain.. Journal of Biological Chemistry.

[32] De Strooper B (2003). Aph-1, Pen-2, and Nicastrin with Presenilin Generate an Active gamma-Secretase Complex.. Neuron.

[33] Borchelt DR, Ratovitski T, Van Lare J, Lee MK, Gonzales V (1997). Accelerated amyloid deposition in the brains of transgenic mice coexpressing mutant presenilin 1 and amyloid precursor proteins.. Neuron.

[34] Li T, Wen H, Brayton C, Laird FM, Ma G (2007). Moderate reduction of gamma-secretase attenuates amyloid burden and limits mechanism-based liabilities.. J Neurosci.

[35] Li T, Wen H, Brayton C, Das P, Smithson LA (2007). Epidermal growth factor receptor and notch pathways participate in the tumor suppressor function of gamma-secretase.. J Biol Chem.

[36] Lai MT, Chen E, Crouthamel MC, DiMuzio-Mower J, Xu M (2003). Presenilin-1 and presenilin-2 exhibit distinct yet overlapping gamma-secretase activities.. J Biol Chem.

[37] Placanica L, Tarassishin L, Yang G, Peethumnongsin E, Kim S (2009). Pen2 and presenilin-1 modulate the dynamic equilibrium of presenilin-1 and presenilin-2 gamma-secretase complexes.. J Biochem.

[38] Cai H, Wang Y, McCarthy D, Wen H, Borchelt DR (2001). BACE1 is the major beta-secretase for generation of Abeta peptides by neurons.. Nat Neurosci.

[39] Borchelt DR, Thinakaran G, Eckman CB, Lee MK, Davenport F (1996). Familial Alzheimer's disease-linked presenilin 1 variants elevate Abeta1-42/1-40 ratio in vitro and in vivo.. Neuron.

[40] Lee MK, Borchelt DR, Kim G, Thinakaran G, Slunt HH (1997). Hyperaccumulation of FAD-linked presenilin 1 variants in vivo.. Nat Med.

[41] Selkoe D, Kopan R (2003). Notch and Presenilin: regulated intramembrane proteolysis links development and degeneration.. Annu Rev Neurosci.

[42] Kumar VB, Franko M, Banks WA, Kasinadhuni P, Farr SA (PS1) levels in senescence-accelerated mice (SAMP8) may indirectly impair memory by affecting amyloid precursor protein (APP) processing.. J Exp Biol.

[43] Meckler X, Roseman J, Das P, Cheng H, Pei S (2010). Reduced Alzheimer's disease ss-amyloid deposition in transgenic mice expressing S-palmitoylation-deficient APH1aL and nicastrin.. J Neurosci.

[44] Steiner H, Winkler E, Edbauer D, Prokop S, Basset G (2002). PEN-2 is an integral component of the gamma-secretase complex required for coordinated expression of presenilin and nicastrin.. J Biol Chem.

[45] Savonenko A, Xu GM, Melnikova T, Morton JL, Gonzales V (2005). Episodic-like memory deficits in the APPswe/PS1dE9 mouse model of Alzheimer's disease: relationships to beta-amyloid deposition and neurotransmitter abnormalities.. Neurobiol Dis.

[46] Ghosh A, Greenberg ME (1995). Distinct roles for bFGF and NT-3 in the regulation of cortical neurogenesis [see comments].. Neuron.

[47] Thinakaran G, Borchelt DR, Lee MK, Slunt HH, Spitzer L (1996). Endoproteolysis of presenilin 1 and accumulation of processed derivatives in vivo.. Neuron.

[48] Luo Wj, Wang H, Li H, Kim BS, Shah S (2003). PEN-2 and APH-1 Coordinately Regulate Proteolytic Processing of Presenilin 1.. Journal of Biological Chemistry.

[49] Li YM, Lai MT, Xu M, Huang Q, DiMuzio-Mower J (2000). Presenilin 1 is linked with gamma-secretase activity in the detergent solubilized state.. Proc Natl Acad Sci U S A.

[50] Xu G, Gonzales V, Borchelt DR (2002). Rapid Detection of Protein Aggregates in the Brains of Alzheimer Patients and Transgenic Mouse Models of Amyloidosis.. Alzheimer Dis Assoc Disord.

[51] Vehmas AK, Borchelt DR, Price DL, McCarthy D, Wills-Karp M (2001). beta-Amyloid peptide vaccination results in marked changes in serum and brain Abeta levels in APPswe/PS1DeltaE9 mice, as detected by SELDI-TOF- based ProteinChip technology.. DNA Cell Biol.

